# Clinical effectiveness of efinaconazole 10% solution for treatment of onychomycosis with longitudinal spikes

**DOI:** 10.1111/1346-8138.16035

**Published:** 2021-07-01

**Authors:** Shinichi Watanabe, Ken Iozumi, Masatoshi Abe, Yoshiko Ito, Takashi Uesugi, Takashi Onoduka, Ichiro Kato, Fumihiro Kato, Kazuo Kodama, Hidetoshi Takahashi, Osamu Takeda, Koki Tomizawa, Yasuki Tateishi, Mizue Fujii, Jun Mayama, Fumio Muramoto, Hidemi Yasuda, Kiyomitsu Yamanaka, Tsunao Oh‐i, Hiroko Kasai, Ryoji Tsuboi, Naoko Hattori, Ryuji Maruyama, Tokuya Omi, Harunari Shimoyama, Ichiro Nakasu, Emiko Watanabe‐Okada, Shuhei Nishimoto, Takashi Mochizuki, Masao Fukuzawa, Mariko Seishima, Kazumitsu Sugiura, Osamu Yamamoto, Masahisa Shindo, Hiroe Kiryu, Masahiro Kusuhara, Motoi Takenaka

**Affiliations:** ^1^ Department of Dermatology Teikyo University School of Medicine Tokyo Japan; ^2^ Department of Dermatology Tokyo Metropolitan Police Hospital Tokyo Japan; ^3^ Sapporo Skin Clinic Sapporo Japan; ^4^ Ito Skin Clinic Sapporo Japan; ^5^ Uesugi Dermatology Clinic Sapporo Japan; ^6^ Asanuma Dermatology Clinic Chitose Japan; ^7^ Eniwa Station Dermatology Clinic Eniwa Japan; ^8^ Kato Dermatology Clinic Sapporo Japan; ^9^ Megumino Dermatologic Clinic Eniwa Japan; ^10^ Takagi Dermatological Clinic Obihiro Japan; ^11^ Takeda Dermatological Skin Care Clinic Sapporo Japan; ^12^ Nopporo Dermatology Clinic Ebetsu Japan; ^13^ Showa Skin Clinic Hakodate Japan; ^14^ Department of Dermatology Asahikawa Medical University Hospital Asahikawa Japan; ^15^ Chitose Dermatology and Plastic Surgery Clinic Chitose, Japan; ^16^ Shinoro Dermatology Clinic Sapporo Japan; ^17^ Fukuzumi Dermatology Clinic Sapporo Japan; ^18^ Yamanaka Skincare Clinic Sapporo Japan; ^19^ Atago Dermatology Clinic Tokyo Japan; ^20^ Department of Dermatology Kitasato University Kitasato Institute Hospital Tokyo Japan; ^21^ Department of Dermatology Tokyo Medical University Tokyo Japan; ^22^ Naoko Dermatology Clinic Tokyo Japan; ^23^ Maruyama Dermatology Clinic Tokyo Japan; ^24^ Queen’s Square Medical Center Yokohama Japan; ^25^ Department of Dermatology Teikyo University Mizonokuchi Hospital Kawasaki Japan; ^26^ Nemunoki Dermatology Clinic Kawasaki Japan; ^27^ Department of Dermatology Saiseikai Yokohama‐shi Tobu Hospital Yokohama Japan; ^28^ Department of Dermatology Saiseikai Kanagawa Hospital Yokohama Japan; ^29^ Department of Dermatology Kawasaki Municipal Hospital Kawasaki Japan; ^30^ Department of Dermatology Kanazawa Medical University Kanazawa Japan; ^31^ Department of Dermatology Ina Central Hospital Ina Japan; ^32^ Department of Dermatology Gifu University Graduate School of Medicine Gifu Japan; ^33^ Department of Dermatology Fujita Health University Toyoake Japan; ^34^ Division of Dermatology Department of Medicine of Sensory and Motor Organs Tottori University Faculty of Medicine Yonago Japan; ^35^ Department of Dermatology National Hospital Organization Hamada Medical Center Hamada Japan; ^36^ Kiryu Dermatology Clinic Fukuoka Japan; ^37^ Kusuhara Dermatology Clinic Fukuoka Japan; ^38^ Department of Dermatology Nagasaki University Hospital Nagasaki Japan

**Keywords:** dermatophytoma, efinaconazole, longitudinal spike, onychomycosis, topical triazole antifungal

## Abstract

Onychomycosis with longitudinal spikes in the nail plate has been reported to be refractory to oral drugs as with dermatophytoma. We evaluated the efficacy of 10% efinaconazole solution in the treatment of onychomycosis with longitudinal spikes. Of the 223 subjects who were enrolled in a previous study, a post‐hoc analysis of 82 subjects with longitudinal spikes was performed in this study. The opacity ratio of longitudinal spikes was decreased over time from 8.1 to 0.9 at the final assessment. In addition, the longitudinal spike disappearance rate increased early after the application to 81.7% at the final assessment. Therefore, 10% efinaconazole solution can be a first‐line drug for longitudinal spikes, which have been regarded as refractory to oral drugs.

## 1 INTRODUCTION

Onychomycosis is a common disease in daily medical practice, and affects approximately 10% of the population in Japan.^1^ The disease types of onychomycosis are classified by the fungal entry route; as per the Japanese guidelines,^2^ they are classified into the following five types: (i) distal and lateral subungual onychomycosis (DLSO); (ii) superficial white onychomycosis; (iii) proximal subungual onychomycosis (PSO); (iv) endonyx onychomycosis; and (v) total dystrophic onychomycosis (TDO). In addition, there may be specific disease types such as onychomycosis with longitudinal spikes (yellow or white linear affected area) or onychomycosis with dermatophytoma (round circumscribed yellow or a white patch) in the nail plate. Recently, DLSO with a V‐shaped affected area at the tip of the nail are called “wedge shape” in Japan. However, it is a normal manifestation in DLSO; thus, it is different from longitudinal spikes.

In daily clinical practice, oral antifungal drugs are effective in many cases. However, longitudinal spikes or dermatophytoma have been reported to be refractory to oral drugs.^3^, ^4^, ^5^, ^6^ Therefore, in previous clinical trials for onychomycosis, these disease types were excluded.^7^, ^8^, ^9^ However, after launching 10% efinaconazole solution (EFCZ) as the first topical therapeutic agent for onychomycosis in Japan, some cases with longitudinal spikes responded to EFCZ.

Using the data of the study by Iozumi et al.^10^ where longitudinal spikes were not excluded, we conducted a post‐hoc analysis of the efficacy of EFCZ in onychomycosis with longitudinal spikes.

## 2 METHODS

### 2.1 Study subjects

This was a retrospective study to investigate the data from the study conducted by Iozumi et al.^10^ after the end of the observation period. An ethical review was conducted at each study site, and approval was obtained. This study was conducted in compliance with the Ethical Guidelines for Medical and Health Research Involving Human Subjects; information on the study including its objectives was notified or disclosed, and opportunities for refusal were secured as much as possible. From the study by Iozumi et al.,^10^ patients who were assessed to have onychomycosis with longitudinal spikes based on the images taken at the start date of the study by both the medical expert and the investigator of the study site were included.

### 2.2 Assessment and fixation of onychomycosis with longitudinal spikes

In this study, onychomycosis with a yellow or white linear affected area was defined as onychomycosis with longitudinal spikes. We defined longitudinal spike as a clinical manifestation of longitudinal linear opacity; however, the fungal histological findings, such as a dense mass of dermatophytes, were not confirmed. The medical expert evaluated whether or not the masked images of onychomycosis at the start of the study corresponded to the definition. After this, the investigator at each site assessed whether the image at the start of the study corresponded to the definition of patients at their site. When the assessments by the medical expert and the investigator were the same, it was placed as the assessment result. If the assessments of the medical expert and the investigator were different, the two parties discussed and the agreed assessment was placed as the assessment result. When multiple longitudinal spikes were observed in the study nail, the one with a higher opacity ratio was evaluated.

The opacity ratio and width ratio of the longitudinal spike were calculated. The opacity ratio was defined by the following formula: [*X*/*Y*] × 10, where *X* and *Y* represented the length of the longitudinal spikes and the length of the entire toenail (distance between free edge and posterior nail fold), respectively. The width ratio was the ratio of the widest of the longitudinal spike to the width of the posterior nail fold. Disappearance was assessed at the time point when the opacity ratio of the longitudinal spike was zero. Each length was measured using Image J (version 1.53a/Java 1.8.0_112).

### 2.3 Primary end‐point

The primary end‐point was change in longitudinal spike opacity ratio and disappearance rate over time.

### 2.4 Secondary end‐points

Evaluation of the complete cure rate, mycological cure rate, and treatment success rate were for the entire infected area, not for longitudinal spikes.

Complete cure was defined as a 0% clinical involvement of the target nail, with a negative KOH examination result.

Mycological cure was defined as a negative result in the KOH examination of the target nail.

Treatment success was defined as a reduction in clinical involvement to 10% or less of the target nail.

Other secondary end‐points were changes over time in the opacity area and in the decrease of the affected nail area.

### 2.5 Evaluation time points

The evaluation time points were at the start of application, weeks 12, 24, 36, 48, 60, and 72, and the final evaluation (week 72 or the date of completion of administration).

In the data to be evaluated in this study, the data from the study conducted by Iozumi et al.^10^ were used for the following:

- Study nail (left or right first toenail): if the right and left first toenails were affected, the one with a larger opacity area was evaluated. If the areas were the same, the right toenail was evaluated.
- Opacity area (%): the opacity area was measured by visual measurement for the percentage of the opaque area (including the area of opacity lost due to nail cutting) with the nail area assumed to be 100%.
- KOH examination: results of the target nail on the evaluation time point after the start of application.
- Fungal culture test: results of the target nail at the start of application.
- Background information: sex, age, opacity area, and causative fungal species.

### 2.6 Statistical analysis

For each end‐point, the number of subjects and proportion (%) were obtained for nominal variables, and summary statistics were calculated for continuous variables. The statistical analysis software R version 3.6.2 (R Core Team) was used. Missing data were imputed using the last observation carried forward method.

## 3 RESULTS

**FIGURE 1:**
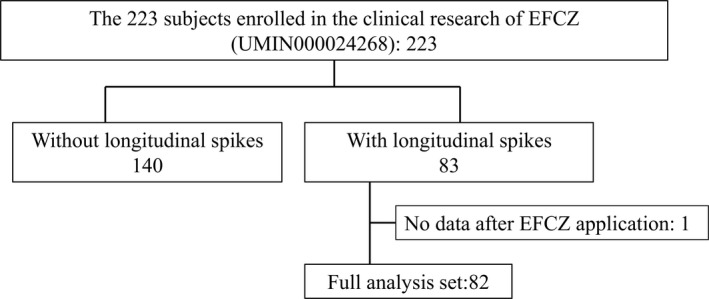
Distribution of the subjects. EFCZ, 10% efinaconazole solution

Figure 1 shows the disposition of the subjects. Of the 223 subjects enrolled in the clinical research of EFCZ (UMIN000024268), 83 patients (37.2%) had longitudinal spikes. Excluding one subject who lacked post‐EFCZ application data, 82 subjects were included as the analysis population. Table 1 shows the subjects’ baseline characteristics. Of the subjects, 49 were male (59.8%) and 33 were female (40.2%), with a mean age of 61.7 ± 11.5 years and 46 subjects (56.1%) aged less than 65 years and 36 subjects (43.9%) aged 65 years or more. The causative fungal species were *Trichophyton rubrum* in 51 subjects (62.2%), *Trichophyton interdigitale* (*Trichophyton mentagrophytes*) in 19 subjects (23.2%), and *Trichophyton* species in 12 subjects (14.6%). The width ratio of the longitudinal spike at baseline was 2.0 (95% confidence interval [CI], 1.7–2.2).

**TABLE 1:** Baseline characteristics

Variables	n = 82
Sex	
Male	49 (59.8)
Female	33 (40.2)
Age	61.7 ± 11.5
<65 years old	46 (56.1)
≥65 years old	36 (43.9)
Target nail for assessment	
Right	46 (56.1)
Left	36 (43.9)
Causative fungal species	
*Trichophyton rubrum*	51 (62.2)
*Trichophyton interdigitale*	19 (23.2)
*Trichophyton* species	12 (14.6)

Note

Data are presented as mean ± standard deviation or number of subjects (proportion [%]).

**FIGURE 2:**
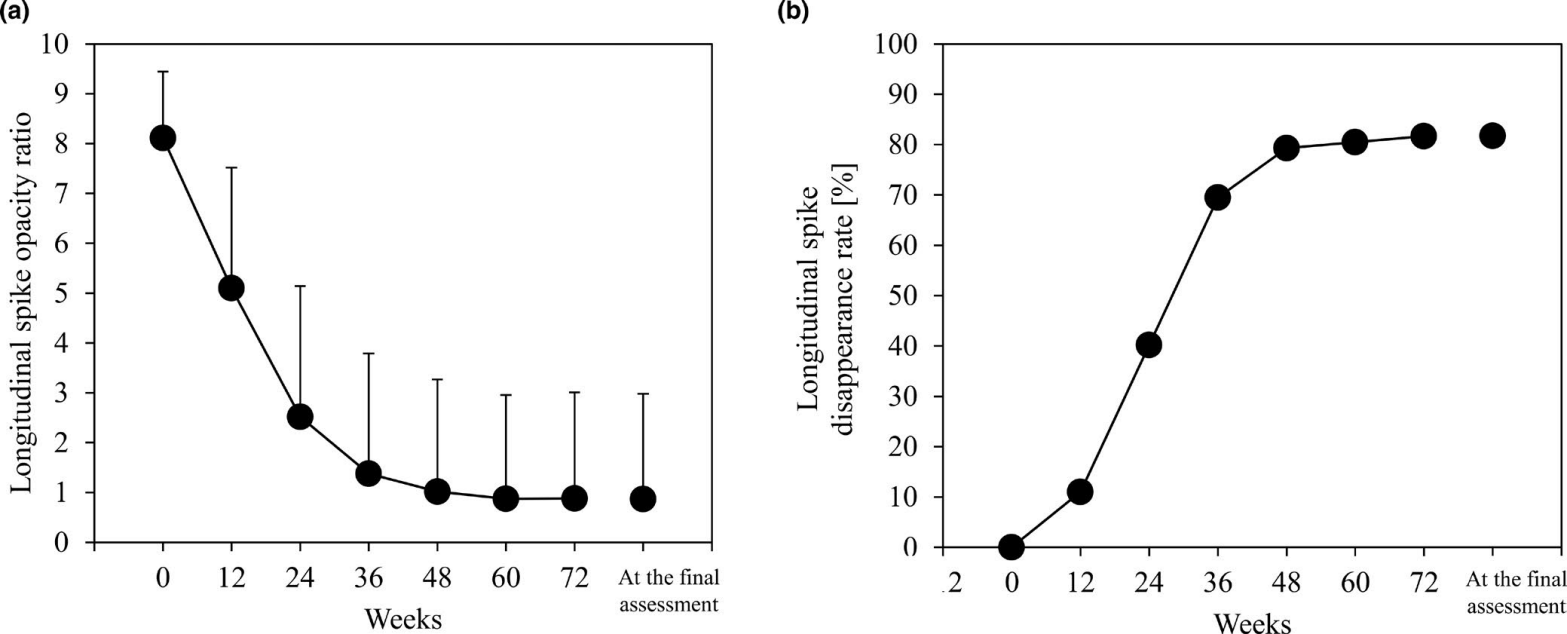
Changes in primary end‐point over time. (a) Opacity ratio of longitudinal spikes. (b) Disappearance rate of longitudinal spikes

Table 2 shows the results of each end‐point at the final assessment. Figure 2 shows changes over time in the opacity ratio of the longitudinal spike and changes in the disappearance rate of the longitudinal spike over time. The opacity ratio of the longitudinal spike was 8.1 (95% CI, 7.8–8.4) at baseline, decreasing from the early stage of treatment. It decreased to 2.5 (95% CI, 1.9–3.1) at week 24, which was approximately 30% of the baseline value, and decreased to 0.9 (95% CI, 0.4–1.3) at the final assessment (Figure 2a). The disappearance rate was 41.3% (nearly half disappeared) at week 24, and 81.7% at the final assessment (Figure 2b).

**TABLE 2:** Results of each end‐point

End‐point	n = 82
Longitudinal spike width ratio	
At baseline	2.0 (1.7–2.2)
Longitudinal spike opacity ratio	
At baseline	8.1 (7.8–8.4)
At final assessment	0.9 (0.4–1.3)
Longitudinal spike disappearance rate	81.7 (67)
Complete cure rate	41.5 (34)
Mycological cure rate	72.0 (59)
Treatment success rate	64.6 (53)

Note

Data are presented as means (upper and lower limits of 95% confidence interval) or proportions (%) (number of subjects). The complete cure rate, mycological cure rate, and treatment success rate presented at final assessment. Evaluation of the complete cure rate, mycological cure rate, and treatment success rate are for the entire infected area, not for longitudinal spikes.

**FIGURE 3:**
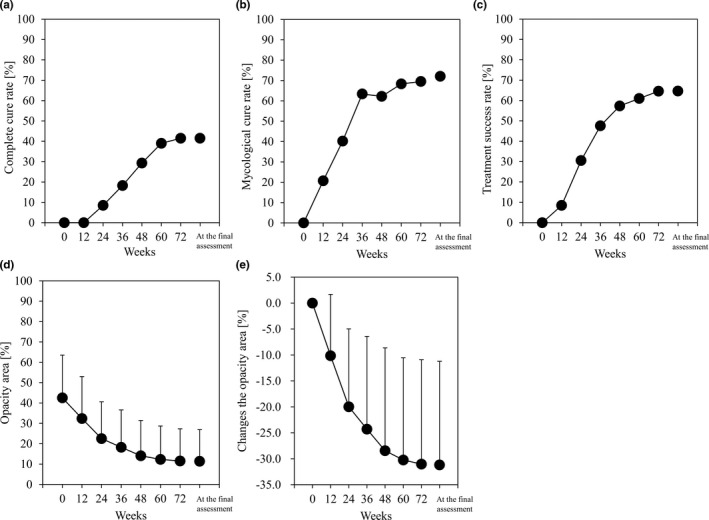
Changes in secondary end‐points over time. (a) Complete cure rate. (b) Mycological cure rate. (c) Treatment success rate. (d) Opacity area. (e) Change in opacity area from baseline

In addition to the evaluation of the longitudinal spike, changes over time in the complete cure, mycological cure, and treatment success rates of the entire opaque part of the target nail are shown in Figure 3. The complete cure rate at the final evaluation was 41.5% (Figure 3a). The mycological cure rate and treatment success rate at the final evaluation were 69.5% and 64.6%, respectively (Figure 3b,c). The total opacity area was 42.5% at baseline and decreased to 11.3% at the final assessment (Figure 3d,e).

**FIGURE 4:**
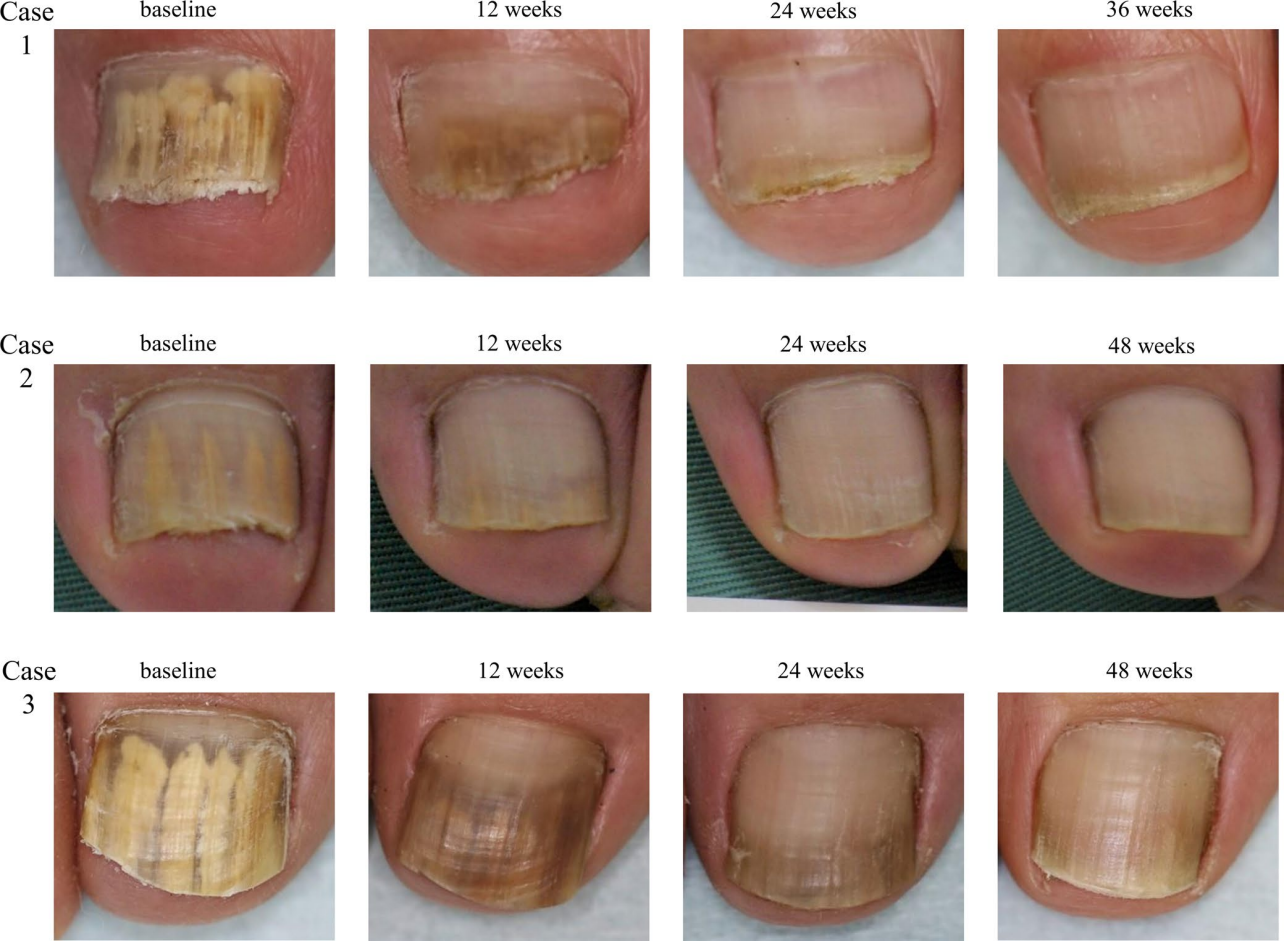
Changes over time in symptoms after application of 10% efinaconazole solution

Representative changes in symptoms after application of EFCZ are presented in Figure 4. Marked improvement was observed; longitudinal spike had disappeared at week 24, and onychomycosis was completely cured at week 36 (subject 1) or week 48 (subjects 2 and 3).

Subgroup analysis was performed for the primary and secondary end‐points. The results are shown in Table 3. In all subgroup analyses, the disappearance rate of the longitudinal spike at the final assessment was comparable to the entire achievement rate. In addition, the rates of complete cure, mycological cure, and treatment success for the entire opacity part of the target nail were all comparable to those of the entire achievement rate.

**TABLE 3:** Subgroup analysis on efficacy end‐points at the final assessment

	Sex		Age		Causative fungal species		
	Male (n = 49)	Female (n = 33)	<65 years (n = 46)	≥65 years (n = 36)	*Trichophyton* *rubrum* (n = 51)	*Trichophyton* *interdigitale* (n = 19)	*Trichophyton* species (n = 12)
Longitudinal spike disappearance rate	79.6 (39)	84.8 (28)	76.1 (35)	88.9 (32)	82.4 (42)	73.7 (14)	91.7 (11)
Complete cure rate	28.6 (14)	60.6 (20)	34.8 (16)	50.0 (18)	27.5 (14)	63.2 (12)	66.7 (8)
Mycological cure rate	69.4 (34)	75.8 (25)	65.2 (30)	80.6 (29)	64.7 (33)	78.9 (15)	91.7 (11)
Treatment success rate	61.2 (30)	69.7 (23)	65.2 (30)	63.9 (23)	52.9 (27)	78.9 (15)	91.7 (11)

Note

Data are presented as percentage (%) (number of subjects). Evaluation of the complete cure rate, mycological cure rate, and treatment success rate are for the entire infected area, not for longitudinal spikes.

## 4 DISCUSSION

Ten percent efinaconazole solution has been shown to be effective for various types of onychomycosis and has been prescribed as a treatment option for onychomycosis. In a global phase 3 study,^7^ EFCZ was shown to be effective for mild to moderate DLSO. Iozumi et al.^10^ evaluated the efficacy of long‐term use of EFCZ for onychomycosis, including severe cases. The complete cure rate at the final evaluation was 31.1%.^10^ In another study, Noguchi et al.^11^ reported an efficacy rate of 65.4% in patients with an infected area exceeding 50%. In 2020, randomized controlled trials^7^, ^12^ of EFCZ with a high level of evidence were cited in the review of local treatment and device treatment for onychomycosis in the Cochrane Library.^13^

In our study, the opacity ratio of the longitudinal spike decreased over time to approximately 1/10th of the baseline value at the final assessment. The disappearance rates were 40.2% at week 24 and 79.3% at week 48, reaching approximately 80%, showing early cure of longitudinal spike. EFCZ was suggested to be a useful option for longitudinal spikes, which had been considered intractable. We consider that EFCZ was effective because a sufficient amount reached the lesion site of longitudinal spikes. Three characteristics of EFCZ can be considered as the reason why a sufficient amount reaches the nail bed: (i) high fungicidal activity in the nail plate and nail with low keratin affinity and excellent nail penetration;^14^ (ii) low surface tension with the inclusion of surfactant;^15^ and (iii) increased drug delivery into infected nails due to low surface tension.^16^

Dermatophytoma is characterized by white or yellow opacities in the center of the nail plate, with the lesion present in the lower layer of the nail plate. Dermatophytoma is characterized by white or yellow opacities in the center of the nail plate, with the lesion present in the lower layer of the nail plate. Histologically, the loculated hyperkeratotic mass appears as a densely packed clump of dermatophyte hyphae, which are thick‐walled and somewhat abnormally presented.^3^ The nail plate is cavitated and the cavity becomes semi‐anaerobic, with fungal elements forming a thick pellicle, which inhibits drug penetration and becomes resistant to treatment.^4^ The longitudinal spikes are linear opacities on the nail plate, and the opacity part has a thin cavity, forming a tunnel. Longitudinal spikes and dermatophytoma are closely related pathologies, and the major difference is whether they are vertically linear or have a wide cavity, which is difficult to distinguish in many cases. Thus, as with dermatophytoma, longitudinal spikes have been reported to be refractory to oral drugs. One of the reasons is that a sufficient amount of the drug does not reach the site of fungal infection.^17^

Ten percent efinaconazole solution has been reported to be effective for dermatophytoma. Wang et al.^18^ applied EFCZ to 19 patients with onychomycosis complicated by dermatophytoma and reported that all patients resolved and did not recur during the study. They believe that an effective therapeutic agent must be able to diffuse through the nail plate and remain at high enough antimicrobial concentrations in the subungual space. Shimoyama et al.^19^ reported that the complete cure rate of dermatophytoma with EFCZ was 60% (3/5). They stated that the reason for the efficacy was that a sufficient concentration of the drug reached the fungal elements in the infected nail due to the specific characteristics of the drug.

According to the data obtained from the study by Iozumi et al.,^10^ the frequency of DLSO with longitudinal spikes was as high as 37.2%. Moreover, part of the infected area remains as longitudinal spikes in many cases when DLSO is treated with oral drugs. EFCZ will also be useful in such cases. As the application of EFCZ can eliminate longitudinal spikes in a short period of time (~6 months), combination therapy with oral drugs will enable complete cure of DLSO in a short period of time.

In the study by Tsunemi,^20^ the selection of drugs for each type and severity of onychomycosis was investigated. As the drug most frequently prescribed for onychomycosis with wedge‐shaped lesions, topical drugs accounted for 54.1%, which was higher than that of oral drugs. However, this selection was based on therapeutic experience, and the efficacy of topical agents for wedge‐shaped lesions was not clear. Strictly speaking, wedge‐shaped lesions are different from longitudinal spikes, but our study results demonstrated that EFCZ is the first‐line therapy for onychomycosis with these lesions based on evidence. On the other hand, since it takes time to cure PSO or TDO with topical agents, treatment should be selected according to the disease type and severity.

Among cases of onychomycosis with longitudinal spikes in this study, 51 (62.2%) of the 82 were caused by *T*. *rubrum*. There was no significant difference in the fungal species reported in the epidemiological studies of onychomycosis in Japan, and it was found that the main causative fungal species was *T*. *rubrum*. For the causative species of dermatophytoma, although the sample size was only seven patients, a study reported that *T*. *rubrum* was detected in three of the seven.^21^ This is the first report on causative fungal species of longitudinal spikes, providing new findings. However, in this study, the sampling site was not limited to the longitudinal spikes, and we cannot rule out the possibility that the causative fungal species of the longitudinal spike were different from those collected from other lesions. Therefore, examination of the causative species localized to the longitudinal spike will be needed in the future.

In conclusion, this study clarified the clinical effectiveness of EFCZ for the treatment of onychomycosis with longitudinal spikes. Since this is a multicenter study with a sample size of over 80 subjects, it will provide important findings to clarify the therapeutic effect. Although data on the efficacy of longitudinal spikes have been insufficient, the results of our study showed that EFCZ has a high therapeutic effect and can be the first‐line therapy.

## CONFLICT OF INTEREST

S.W. received a consultancy fee from Kaken Pharmaceutical, and T.O. has stock in Kaken Pharmaceutical.
